# Scott Blair Fractional-Type Viscoelastic Behavior of Thermoplastic Polyurethane

**DOI:** 10.3390/polym15183770

**Published:** 2023-09-14

**Authors:** Christian Pichler, Stefan Oberparleiter, Roman Lackner

**Affiliations:** Material Technology Innsbruck, University of Innsbruck, Technikerstraße 13, A-6020 Innsbruck, Austria; stefan.oberparleiter@uibk.ac.at (S.O.); roman.lackner@uibk.ac.at (R.L.)

**Keywords:** power-law creep, logarithmic creep, Lomnitz model, hydrogen bonds

## Abstract

In this paper, the experimental characterization of the viscoelastic properties of thermoplastic polyurethane (TPU) samples through creep experiments is presented. Experiments were conducted at different constant temperature levels (15, 25, and 35 ${}^{\circ }$C), for three different tensile stress levels (0.3, 0.5, and 0.7 MPa), and at different physisorbed water contents, providing access to: (i) the temperature dependency of creep parameters and (ii) the assessment, if behavior is indeed viscoelastic. The physisorbed water content was achieved by exposing virgin samples to environments with relative humidity ranging from 0 to 80 percent until mass stability was reached. Creep tests were conducted immediately afterwards with this particular humidity level. The main results of this study are as follows. The temperature dependency of the obtained creep parameters is well described in Arrhenius plots. With regard to water content, two prototype material responses were observed in the experimental program and accurately modeled using the following fractional-type models: (i) Scott Blair-type (i.e., power-law-type) only behavior, pronounced for the combination of low water content/low temperature; (ii) combined Scott Blair plus Lomnitz (i.e., log-type) behavior for high water content/high temperature. This change in behavior associated with certain thresholds for the specified environmental conditions (temperature and relative humidity) may indicate the initiation of hydrogen bond breakage and rearrangement (carbamate H-bonds and physisorbed water H-bonds). Regarding the short-term or quasi-instantaneous behavior, the Scott Blair element seems highly appropriate and may be better suited than the standard elastic model: the Hookean spring. We associated Scott Blair behavior with the load-induced, quasi-instantaneous re-arrangement of polymer network chains. The secondary viscoelastic mechanism associated with the Lomnitz element, hydrogen bond breakage and rearrangement, comes into play for higher temperatures and/or higher physisorbed water contents. In this case, the contribution of the two constitutive elements is well separated due to the large number of the characteristic time of the Lomnitz element, much larger than the respective value for the Scott Blair element.

## 1. Introduction

**Figure 1:**
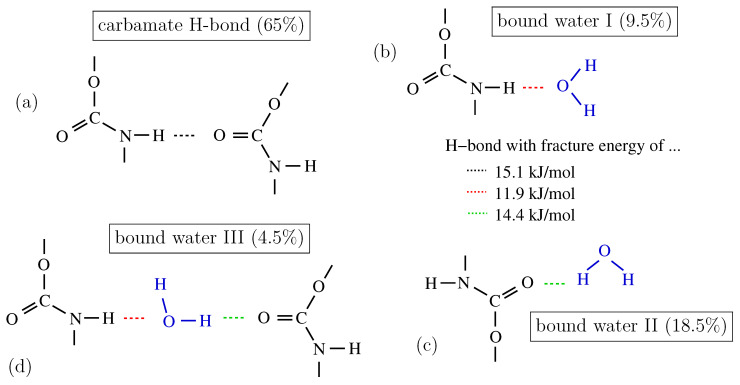
Hydrogen bonds in the molecular dynamics (MDs) model system containing the initial three methylene diphenyl dicarbamate (MDC) molecules and one water molecule (numbers in parentheses represent the prevalence found in a total of 600,000 MDs analyses) [2]: (**a**) anhydrous urethane hydrogen bond; (**b**–**d**) the most prevalent hydrogen bonds involving the water molecule. Two more hydrogen bond types involving the water molecule were obtained by Xu et al. [2]: two hydrogen bonds toward the double-bound oxygen atom in two MDC molecules (bound water IV, a fracture energy of 14.4 + 14.4 kJ/mol, a prevalence of 2.1%), three hydrogen bonds toward the oxygen atom in two MDC molecules and one toward the secondary amine in the third MDC molecule (V, 2 × 14.4 + 11.9 kJ/mol, 0.4%).

Thermoplastic polyurethanes (TPUs) are defined as block copolymers created through the synthesis of diisocyanates, polyols, and chain extenders. TPUs can be classified as either (i) polyester-based or (ii) polyether-based polyurethanes. As TPUs are frequently applied in high-humidity or even hypodermic environments [1], calibrated engineering models quantitatively describing the thermo-hygro-mechanical behavior of TPUs may play a critical role in anticipating durability and long-term stability within industrial and structural uses. While the long-term exposure (spanning several months or years) of TPUs to water results in chemical aging, involving network chain cleavage, the short-term interaction of TPUs with water can be described as physisorption (physisorption is a process in which the electronic structure of the atom or molecule is barely perturbed upon adsorption, i.e., associated with London forces, dipole/dipole interaction, induced dipole/dipole interaction, etc. and hydrogen bonds; the latter interaction may be the dominant physisorption mechanism in the investigated material). Hereby, urethane hydrogen bonds (as shown in Figure 1a, modified from Xu et al. [2]) are converted to urethane–water–urethane hydrogen bonds (Figure 1b–d). The establishment of these urethane–water–urethane hydrogen bonds is accompanied by a decrease in physical interactions between network chains. Thus, hydrophilic segments are solvated, which may substantially alter the micro-mechanical characteristics of the polymer [3]. The short-term physisorption behavior of the material employed in the present study has been summarized in [4]; see particularly the sorption isotherm (Figure 3 in [4]).

In this paper, we focus on the mechanical behavior in response to service loads (the strength of the material, associated with ultimate loading, is not the focus of this paper); hence, creep experiments were conducted immediately after the thermo-hygral conditioning of virgin samples (for sample preparation see Section 2). The creep tests were performed at the same thermo-hygral conditions: constant temperature and constant humidity in the sample-enclosing environment.

For the time-dependent mechanical characterization at low stress levels (as compared to material strength), one may conduct (i) creep experiments—sample loading is maintained with stress $\sigma \left(t\right)=\sigma_{0}$ = const., or (ii) relaxation experiments—sample deformation kept constant with strain $\varepsilon \left(t\right)=\varepsilon_{0}$ = const.; the complimentary value is monitored: strain history ε(t) in a creep test, stress relaxation history σ(t) otherwise. Both types of experiments are usually conducted at stress levels significantly smaller than the strength of the material, i.e., σ≪ strength. When (i) scaling the monitored evolution as $J\left(t\right)=\varepsilon \left(t\right)/\sigma_{0}$ for the creep test (or $R\left(t\right)=\sigma \left(t\right)/\varepsilon_{0}$ for the relaxation test) and (ii) in case the obtained creep compliance function J(t) (or the relaxation function R(t)) is independent on stress $\sigma_{0}$ (on strain $\varepsilon_{0}$), then, the material behavior falls within the realm of *linear* viscoelasticity. For linear viscoelastic materials, one may predict the relaxation behavior from the measured creep behavior, or vice versa, as the Laplace–Carson transformed creep and relaxation functions, respectively, which are inter-related as L[J(t)]=1/L[R(t)] (see Appendix A).

The principal objective of this study is two-fold: (i) The identification of the most appropriate viscoelastic material model(s) for a consistent representation of data in the investigated experimental envelope (environmental conditions and level of loading). Hereby, we strive for models with a rather low number of adjustable material parameters. This thus opens (ii) the possibility to interpret these very parameters with regard to surmised microstructural mechanisms, which cause the observed macroscopic behavior.

In the course of the presented work, we extensively make use of pertinent models from so-called fractional viscoelasticity [5,6,7,8,9]: a logarithmic-type creep model, the so-called Lomnitz model [10,11,12,13,14] and a power-law type creep (and relaxation) model, the so-called Scott Blair model [15,16,17,18]. With regard to the previous applications of these models toward polyurethane- and polyurethane-based materials see, e.g., [19,20,21,22].

## 2. Material and Sample Preparation

We investigated an aromatic, polyether-based TPU, trading under the name “Lubrizol Estane 58887 TPU” (lot number 02467796AS), characterized by a Shore A hardness of 87. (lubrizol.com, accessed on 12 July 2023) $T_{g}$ (characterized by the manufacturer, differential scanning calorimetry) amounts to −45 ${}^{\circ }$C, and the melting temperature to 140 ${}^{\circ }$C, respectively. As the experimental campaign summarized in this paper was conducted in the temperature range of 15 to 35 ${}^{\circ }$C, the polymer was investigated in the rubbery state.

Prior to sample preparation, the designated granular TPUs, as provided by the manufacturer, was preconditioned by storing in a drying cabinet at 100 ${}^{\circ }$C for two hours. Disc-shaped specimens (diameter of 30 mm, thickness of approximately 1 mm) were assembled with a Struers Citopress-15 equipped with a 30 mm mounting unit, a compression of 300 bars at 180 ${}^{\circ }$C for 6 min, followed by 5 min water cooling. Strip-shaped specimens (length of 25 mm, width of 10 mm) were cut off the discs. As, in this paper, we focus on the short-term viscoelastic behavior, the strip-shaped samples were stored (until mass and temperature stability are reached) at the defined thermo-hygral conditions, i.e., constant temperature *T* and sample enclosing humidity *h*, to be used in the subsequent creep experiments. Based on our previous investigation of hyrgral properties of TPUs [4], the physisorbed water content related to *h* is summarized in Table 1.

## 3. Constitutive Models for Viscoelastic Characterization of Time-Dependent Behavior

**Figure 2:**
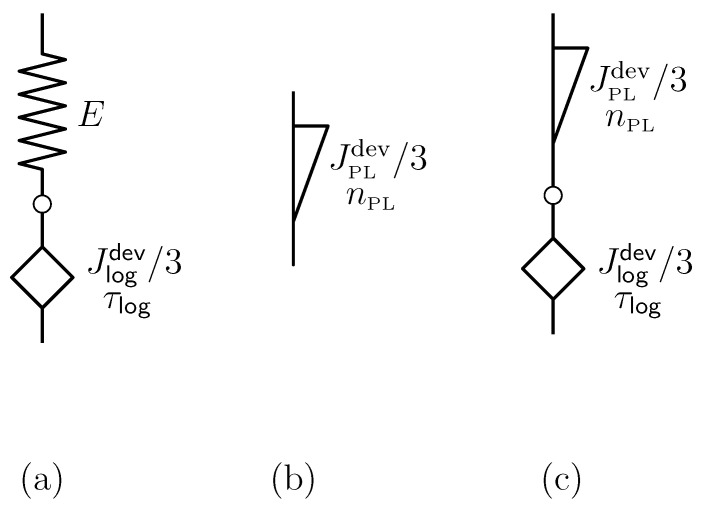
Viscoelastic material models employed in this paper: (**a**) Hookean spring connected in series with Lomnitz element, (**b**) Scott Blair element, frequently denoted as “springpot” in the literature, and (**c**) Scott Blair element connected in series with Lomnitz element.

We will employ two viscoelastic material models, which have proven to effectively represent the time-dependent deformation/stress history of polyurethane foams [23] with a minimum number of material parameters to be determined: (i) Lomnitz element and (ii) Scott Blair element, separately, connected in series, or connected with a Hookean spring, modeling instantaneous and fully recoverable material response (see Figure 2).

Both the Lomnitz element and Scott Blair element fall within the class of so-called fractional viscoelasticity [5,6,7,8,9] with a superior performance as compared to classical models (Maxwell model, Kelvin–Voigt model, and other in parallel/in series arrangements of Hookean springs and (linear) Newtonian dampers) and are able to represent the compliance or relaxation function with a significantly smaller number of parameters [24], as compared to so-called chain models (e.g., the assembly of a large number of Maxwell or Kelvin–Voigt elements). Striving for the physical interpretation of material parameters is a substantial argument for the reduction in the number of parameters to be determined from a dataset. Furthermore, with increasing numbers of material parameters, the proof of uniqueness of the back-calculated parameter set becomes either increasingly difficult or all but possible.

### 3.1. Consideration of a Loading Ramp for Analysis of Creep Test

In this paper, we conducted creep experiments for the determination of the creep compliance function. With regard to the theoretical case of a perfect uniaxial creep experiment, one considers the instantaneous uniaxial loading of a slender sample with the application of a constant compressive stress $\sigma \left(t\right)=H\left(t\right)\sigma_{0}$, with H(t) denoting the Heaviside step function and the monitoring of the time-dependent strain in the direction of loading ε(t). This results in the uniaxial compliance for t>0 as

(1) $$J\left(t\right)=\frac{\varepsilon (t)}{\sigma_{0}}.$$

Practically, such an instantaneous load application is not accomplishable, and some finite duration is required. Usually, stress is applied in the form of a loading ramp, $\sigma =\sigma_{0}t/t_{0}$, for $0\leq t\leq t_{0}$, and held constant, $\sigma =\sigma_{0}$ = const., for $t>t_{0}$, during the so-called dwelling phase of the creep experiment. By liaising experimental data and the previously defined compliance function in the case of a linear viscoelastic material, the strain history can be obtained from the infinitesimal form of the Boltzmann superposition principle:

(2) $$\varepsilon \left(t\right)=\int_{0}^{t}J\left(t-\tau \right)d\sigma \left(\tau \right).$$

Here, for $t\leq t_{0}$, the strain history follows from $d\sigma /d\tau =\sigma_{0}/t_{0}$ as

(3) $$\varepsilon \left(t\leq t_{0}\right)=\frac{\sigma_{0}}{t_{0}}\int_{0}^{t}J\left(t-\tau \right)d\tau .$$

For $t\geq t_{0}$, the reasoning above leads to

(4) $$\varepsilon \left(t\geq t_{0}\right)=\frac{\sigma_{0}}{t_{0}}\int_{0}^{t}J\left(t-\tau \right)d\tau -\frac{\sigma_{0}}{t_{0}}\int_{t_{0}}^{t}J\left(t-\tau \right)d\tau ,$$

where

(5) $$\sigma_{0}\left(t\geq t_{0}\right)=\sigma_{0}\frac{t}{t_{0}}-\sigma_{0}\frac{t-t_{0}}{t_{0}}$$

has been used. Bearing in mind the intended backcalculation of material parameters from experimental data, we will denote the strain histories as represented by Equation (3) and Equation (4), respectively, scaled by the target stress $\sigma_{0}$, as the so-called “ramp compliance”

(6) $$\bar{J}=\frac{\varepsilon (t)}{\sigma_{0}}$$

during both the loading phase and the dwelling phase of the creep experiment, respectively, throughout this manuscript.

Note, furthermore, we suppose that the creep process is caused by the deviatoric stress component only. Hence, volumetric creep is neglected, or, more specifically we underline that deviatoric creep compliance is much bigger than the volumetric creep compliance. The uniaxial elastic compliance 1/E, with *E* (MPa) denoting Young’s modulus can also be split into volumetric and deviatoric parts, 1/E=1/3×1/μ+1/9×1/K, with μ and *K* denoting shear and bulk moduli, respectively. In this regard, the uniaxial viscoelastic compliance, denoted as J(t) in this paper, can also be written as a sum of deviatoric and volumetric compliance, respectively, $J=1/3\times J^{dev}+1/9\times J^{vol}$, with volumetric compliance $J^{vol}$ restricted to the elastic one. This split provides the reasoning for the use of the prefactor of 1/3 and the superscript “dev” for the viscous material parameter employed in the analytical uniaxial compliance functions employed for parameter identification.

### 3.2. Lomnitz Element

**Figure 3:**
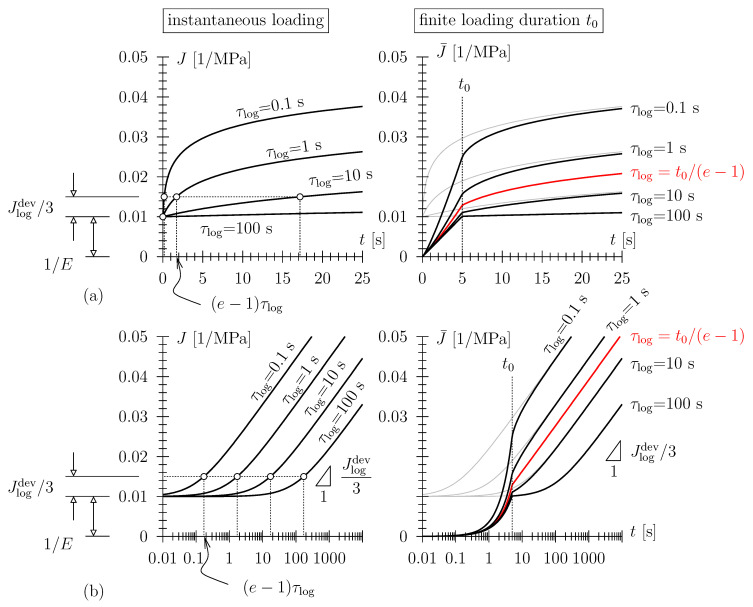
(**left**) Creep compliance $J=1/E+J_{log}^{dev}\ln\left(1+t/\tau_{log}\right)$ (**a**) in linear scale and (**b**) in log-linear scale of Lomnitz element in series with a Hookean spring with *E* = 100 MPa and $J_{log}^{dev}$ = 0.015 MPa${}^{-1}$; (**right**) the so-called ramp compliance $\bar{J}=\varepsilon \left(t\right)/\sigma_{0}$ in creep test comprising (i) a loading ramp with duration $t_{0}$ = 5 s and constant loading rate and (ii) the dwelling phase with constant stress $\sigma \left(t\geq t_{0}\right)=\sigma_{0}$ = const. (note that in the r.h.s., the ramp compliance has been superimposed with the compliance function (in gray) for better comparability).

In case the (long-term) strain history is proportional to the logarithm of time, i.e., ε(t)∝lnt (indicating that the strain rate is $\dot{\varepsilon }\propto t^{-1}$), the logarithmic or Lomnitz creep compliance function reads:

(7) $$J\left(t\right)=\frac{1}{E}+\frac{1}{3}J_{log}^{dev}\ln\left(1+\frac{t}{\tau_{log}}\right)$$

where the log argument has been made dimensionless, positive, and finite. Furthermore, a Hookean spring, with an elastic uniaxial compliance given as 1/E, has been connected in series with the Lomnitz element. In Equation (7), $J_{log}^{dev}$ (MPa${}^{-1}$) is the logarithmic creep compliance parameter and $\tau_{log}$ (s) the characteristic time of the creep process. In a log-linear diagram, the long-term response of J(t) is characterized by a slope of $J_{log}^{dev}/3$ (see Figure 3b (left)). The Lomnitz model has been employed for the modeling of the viscoelastic behavior of a wide range of natural and man-made materials, including wood, cement-based materials, and polyurethane foam [23,25,26,27,28,29,30]. A shortfall of the Lomnitz model is constituted by the fact that the relaxation function corresponding to J(t) (Equation (7)) may not be obtained analytically [31,32,33]; one has to rely on numerical methods for inverse Laplace–Carson transformation to obtain R(t) pointwise; for details see [23,32,33]. This need for numerical inverse transformation is the reason why we chose creep tests (and not relaxation tests) in the experimental campaign presented in this paper.

For a loading ramp as described above, the ramp compliance is obtained as

(8) $$\bar{J}\left(t\leq t_{0}\right)=\frac{\varepsilon (t)}{\sigma_{0}}=\frac{\sigma_{0}}{E}\frac{t}{t_{0}}+\frac{1}{3}J_{log}^{dev}\left(\frac{t+\tau_{log}}{t_{0}}\ln\left(1+\frac{t}{\tau_{log}}\right)-\frac{t}{t_{0}}\right)$$

and

(9) $$\begin{array}{ccc} \bar{J}\left(t\geq t_{0}\right)=\frac{\varepsilon (t)}{\sigma_{0}} & = & \frac{1}{E}+\frac{1}{3}J_{log}^{dev}\left(\frac{t+\tau_{log}}{t_{0}}\ln\left(1+\frac{t}{\tau_{log}}\right)-\right.\\ \; & \; & \left.\frac{t-t_{0}+\tau_{log}}{t_{0}}\ln\left(1+\frac{t-t_{0}}{\tau_{log}}\right)-1\right), \end{array}$$

respectively. For $t_{0}\to 0$ follows $\lim_{t_{0}\to 0}\varepsilon \left(t\right)/\sigma_{0}=1/E+1/3J_{log}^{dev}\ln\left(1+t/\tau_{log}\right)$, i.e., the logarithmic compliance (Equation (7)) previously prescribed. Note, again, that in a log-linear diagram (Figure 3b (right)), the long-term gradient of the model response is given as $1/3J_{log}^{dev}$. Further, note that during the dwelling phase of the creep test, $\bar{J}\left(t\right)$ may either be concave for values of $\tau_{log}$ smaller than (or convex for values of $\tau_{log}$ larger than) the scaled loading duration $t_{0}/\left(e-1\right)=0.582\;t_{0}$ (see Figure 3b (right)).

### 3.3. Scott Blair Element

In [23], we concluded that for closed cell polyurethane foam, the Lomnitz model (Equation (7), Hookean spring in series with a Lomnitz element) well describes the viscoelastic response for a loading duration exceeding approximately 100 s with only an approximate representation of data in the vicinity of $t_{0}$. For the short-term response (loading duration smaller than approximately 100 s), a viscous-only power-law-type compliance approximated the experimental data better. Hereby, we employed the so-called Scott Blair element, also denoted as fractional damper

(10) $$J\left(t\right)=\frac{1}{3}\frac{J_{\mathrm{PL}}^{dev}}{\Gamma [1+n_{\mathrm{PL}}]}{\left(\frac{t}{\tau_{\mathrm{PL}}}\right)}^{n_{\mathrm{PL}}},$$

with material parameters $J_{\mathrm{PL}}^{dev}$ (MPa${}^{-1}$), the power-law compliance parameter and $n_{\mathrm{PL}}$ [–], the power-law exponent; $\tau_{\mathrm{PL}}$ (s) is an arbitrarily chosen time constant which makes the bracket term non-dimensional and was set to 10 s in this paper. Note that the root-like function for $0<n_{\mathrm{PL}}<$1, with an ever decreasing creep rate, yields a compliance characterized by a vertical tangent at t=0, i.e., $\dot{J}\left(t\to 0\right)\to \infty$, i.e., there is a quasi-instantaneous response upon a change of applied stress depicted by this constitutive element, which rendered the introduction of a Hookean spring in the compliance function unnecessary [23]. Note that the introduction of the gamma function prefactor $1/\Gamma [1+n_{\mathrm{PL}}]$ in Equation (10) seems rather arbitrary at first sight. This becomes obvious when regarding the relaxation function of the Scott Blair element,

(11) $$R\left(t\right)=\frac{1}{\Gamma [1-n_{\mathrm{PL}}]}\frac{3}{J_{\mathrm{PL}}^{dev}}{\left(\frac{t}{\tau_{\mathrm{PL}}}\right)}^{-n_{\mathrm{PL}}},$$

which is, as opposed to the Lomnitz element, obtained analytically. Further note that the Scott Blair element reduces to a Newtonian damper with a viscosity of $\tau_{\mathrm{PL}}/J_{\mathrm{PL}}^{dev}$ for $n_{\mathrm{PL}}$ = 1 and Γ[1+1]=Γ[2]=1. For $n_{\mathrm{PL}}\to 0$, a Hookean spring is recovered.

The consideration of the previously defined loading ramp, employing Equations (3) and (4), yields

(12) $$\bar{J}\left(t\leq t_{0}\right)=\frac{\varepsilon (t)}{\sigma_{0}}=\frac{1}{3}\frac{J_{\mathrm{PL}}^{dev}}{\left(1+n_{\mathrm{PL}}\right)\Gamma \left[1+n_{\mathrm{PL}}\right]}\frac{t}{t_{0}}{\left(\frac{t}{\tau_{\mathrm{PL}}}\right)}^{n_{\mathrm{PL}}}$$

and

(13) $$\begin{array}{ccc} \bar{J}\left(t\geq t_{0}\right)=\frac{\varepsilon (t)}{\sigma_{0}} & = & \frac{1}{3}\frac{J_{\mathrm{PL}}^{dev}}{\left(1+n_{\mathrm{PL}}\right)\Gamma \left[1+n_{\mathrm{PL}}\right]}\left[\frac{t}{t_{0}}{\left(\frac{t}{\tau_{\mathrm{PL}}}\right)}^{n_{\mathrm{PL}}}-\right.\\ \; & \; & \left.\frac{t-t_{0}}{t_{0}}{\left(\frac{t-t_{0}}{\tau_{\mathrm{PL}}}\right)}^{n_{\mathrm{PL}}}\right]. \end{array}$$

**Figure 4:**
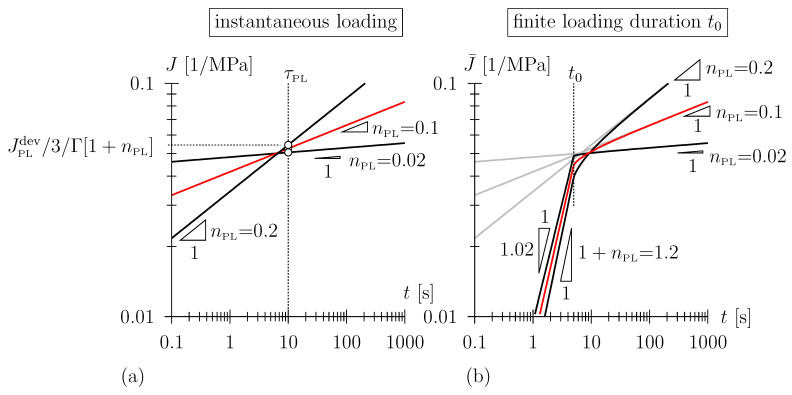
(**a**) Creep compliance $J=1/3J_{\mathrm{PL}}^{dev}/\Gamma \left[1+n_{\mathrm{PL}}\right]{\left(t/\tau_{\mathrm{PL}}\right)}^{n_{\mathrm{PL}}}$ (in double-log scale) of Scott Blair element with $J_{\mathrm{PL}}^{dev}$ = 0.15 MPa${}^{-1}$ and $\tau_{\mathrm{PL}}$ = 10 s; (**b**) ramp compliance $\bar{J}=\varepsilon \left(t\right)/\sigma_{0}$ considering finite loading duration $t_{0}$ = 5 s (note that in (**b**), the ramp compliance has been superimposed with the compliance function (in gray) for better comparability).

Reflecting the power-law nature of the Scott Blair element, the natural choice for the depiction of the compliance function and ramp compliance is a log–log diagram, with J(t) characterized by a constant slope of $n_{\mathrm{PL}}$ (see Figure 4a). The ramp compliance $\bar{J}\left(t\right)$ is characterized by a constant gradient of $\left(1+n_{\mathrm{PL}}\right)$ during the loading phase; the long-term gradient of $\bar{J}\left(t\right)$ is given as $n_{\mathrm{PL}}$ (see Figure 4b).

With regard to parameter identification for the Scott Blair element (and for the other constitutive elements outlined in this paper) from experimental data, either a least-squares fitting of either parts or the entire dwelling phase with the appropriate non-linear fitting function, i.e., for the Scott Blair element with Equation (13), was performed. Material parameters, for the Scott Blair element $J_{\mathrm{PL}}^{dev}$ and $n_{\mathrm{PL}}$, were simultaneously determined by the Levenberg–Marquardt algorithm [34,35]. Within the Levenberg–Marquardt algorithm, the derivatives of the fitting function with respect to the parameters are needed.

## 4. Uniaxial Tensile Creep Experiments

Creep experiments were conducted:

- For three different target stresses of $\sigma_{0}$ = 0.3, 0.5, and 0.7 MPa = const.;
- With a loading ramp characterized by a constant rate of an applied stress of $\dot{\sigma }$ = 0.1 MPa/s, hence the loading duration can be determined as $t_{0}=\sigma_{0}/\dot{\sigma }$;
- With a duration of the dwelling phase of 5000 s;
- At three different temperature levels, at 15, 25, and 35 ${}^{\circ }$C;
- At sample mass equilibrium associated to different sample enclosing humidities of *h* = 0, 40, 60, and 80%, resulting in the physisorbed water contents given in Table 1.

**Figure 5:**
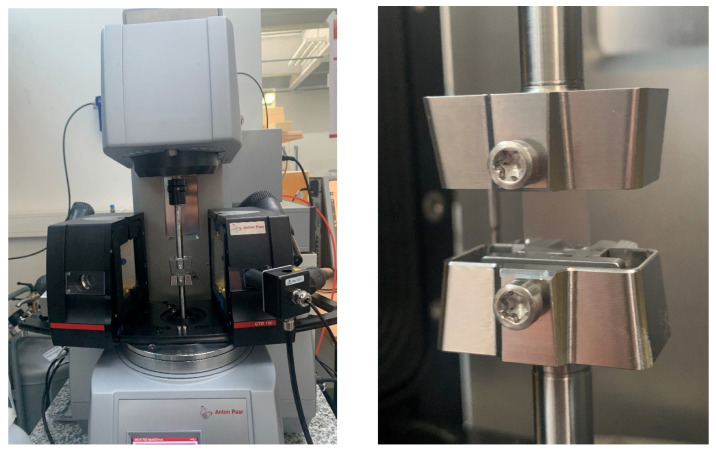
Testing rig with environmental chamber and sample mount employed for the conduction of uniaxial tensile creep experiments.

For this purpose, we employed an Anton Paar MCR 702 rheometer with a linear drive. The sample surrounding atmosphere was assigned within an Anton Paar CTD 180 environmental chamber combined with a Pro Umid MHG100 humidity generator (see Figure 5). The strip-shaped samples were mounted with a rectangular fixture available from Anton Paar. Specimen dimensions were measured prior to each experiment in order to specify the creep load corresponding to the mentioned target stress $\sigma_{0}$.

## 5. Results

Note that the samples were (i) in thermal equilibrium and (ii) at physisorbed mass equilibrium associated with the sample enclosing humidity via the thermostatically and humidity-controlled sample chamber. Hence, possible coupling effects from combined thermo-mechano-sorptive loading/transport processes were circumvented in the experimental program. Such mechano-sorptive coupling effects are well-described in various engineering materials and, e.g., termed the “Pickett effect”, in cement-based materials and mechano-sorptive creep in wood (see Appendix A).

**Figure 6:**
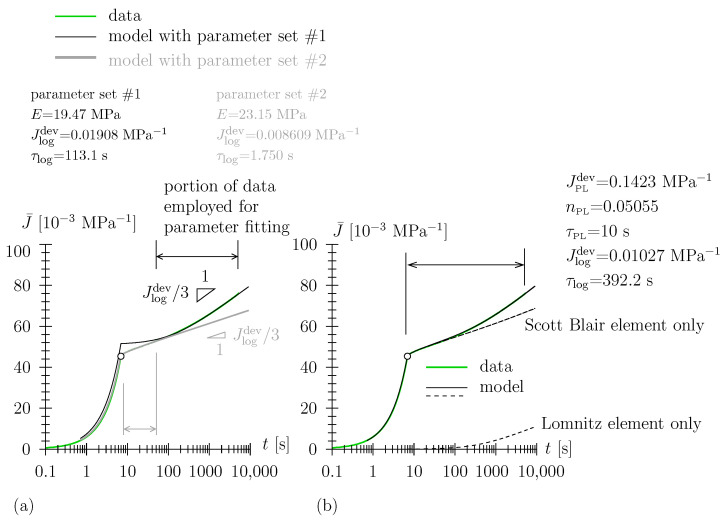
Prototype combined Lomnitz/Scott Blair behavior for high temperature or high humidity experiments, for example, with *h* = 80%, *T* = 35 ${}^{\circ }$C, $\sigma_{0}$ = 0.7 MPa: (**a**) modeling with Lomnitz element in series with a Hookean spring (**b**); modeling with Lomnitz element in series with a Scott Blair element.

**Figure 7:**
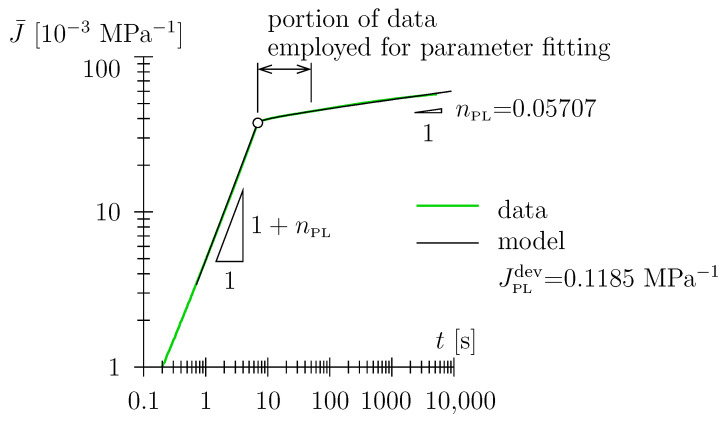
Prototype Scott Blair-only behavior for low humidity and low (or medium)-temperature experiments, for example, with *h* = 0, *T* = 25 ${}^{\circ }$C, $\sigma_{0}$ = 0.7 MPa, modeling with Scott Blair element.

Among the range of investigated humidities (i.e., physisorbed water contents), temperatures, and stress levels, experimental response fell within two prototypical shapes of $\bar{J}\left(t\right)$ (see Figure 6 and Figure 7), with these shapes readily observable when the data are depicted in log-linear or in double-log scale.

For our intial trial, we customized the Lomnitz model (Lomnitz element in series with a Hookean spring) for the representation of $\bar{J}\left(t\right)$ (see Figure 6a). However, for this constitutive model, one is only able to represent either (i) the long-term part of the dwelling phase (parameter set #1) —We have also experienced this better representation of long-term data for polyurethane foam [23]. Note, however, that the notation “long-term” may be misleading here. Due to the log scale of the abscissa *t*, only a short period during and after loading (in the order of $5\times t_{0}$) is not represented very well. In case the engineering application of TPU is quasi-static without short-term (order of one minute) loading fluctuations, the Lomnitz element in series with a Hookean spring is a well-suited constitutive model—or (ii) data in the vicinity of the loading ramp $t\approx t_{0}$ (parameter set #2), i.e., the experimentally observed double-bend shape of $\bar{J}\left(t\right)$ in the dwelling phase cannot be represented. In [23], we already reckoned that a Hookean spring may not be the most suitable constitutive element for the description of the very-short-term material response (we then argued based on the uniqueness of the backcalculated parameter set). When replacing the Hookean spring by a Scott Blair element, i.e., when considering the latter in series with the Lomnitz element, the whole data range, i.e., loading phase and the entire dwelling phase, is represented accurately (see Figure 6b). Furthermore, the contribution of the two constitutive elements is well separated (see dashed graphs in Figure 6b) due to the large number of the characteristic time of the Lomnitz element, $\tau_{log}$, being much greater than the loading duration $t_{0}$. In the investigated range of environmental conditions, the combined Lomnitz/Scott Blair-type response has been identified mostly for the districts of either high humidities or high temperatures (see Table 2 and Appendix B).

On the other hand, for the range of environmental conditions that combine low humidity with low (or medium) temperatures (see Table 2), a solitary Scott Blair constitutive element is sufficient to model the entire data range (see Figure 7). This prototype behavior becomes apparent at a glance when data are plotted in a double-log diagram and linearizes for (i) the loading phase and (ii) the latter part of the dwelling phase. The power-law exponent $n_{\mathrm{PL}}$ is explicitly readable in this type of diagram (see Figure 7). This power-law-type behavior, with a linear relation between the logarithm of compliance and the logarithm of time has frequently been observed in creep experiments on polyurethane in the literature, see, e.g., [36,37]. This is consistent with the observation of a linear relation between the logarithm of stress and the logarithm of time in relaxation experiments, see, e.g., [38] (see Section 3.3).

## 6. Discussion

**Figure 8:**
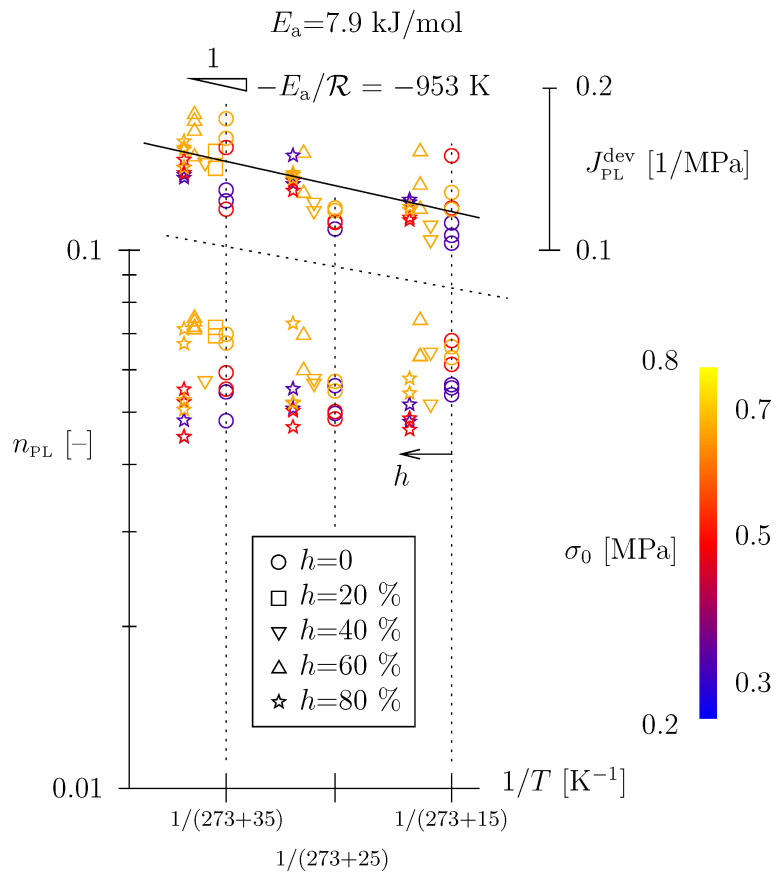
Arrhenius diagram of back-calculated Scott Blair parameters, $\tau_{\mathrm{PL}}$ = 10 s = const. (for better readability h>0 data were slightly offset to the left); the trend line shown for $J_{\mathrm{PL}}^{dev}$, with $-E_{a}/R$ = −953 K, is characterized by a (squared) linear correlation coefficient $r^{2}$ = 0.42; if only *h* = 80% data are taken into account, $-E_{a}/R$ = −880 K with $r^{2}$ = 0.74.

Hence, regardless of the temperature or humidity state, the viscous-only Scott Blair element seems highly appropriate to model the short-term response of TPU and performs qualitatively better than the standard Hookean spring. Figure 8 shows the temperature dependency of the back-calculated Scott Blair parameters. First, there seems to be no influence of stress level, meaning material behavior is indeed within the realm of linear viscoelasticity, as supposed above.

The power-law exponent $n_{\mathrm{PL}}$ is not influenced by the temperature level and amounts to the range $n_{\mathrm{PL}}\approx$ 0.05 to 0.07. $J_{\mathrm{PL}}^{dev}$ shows a temperature dependency well described by an Arrhenius term $\exp[-E_{a}/\left(RT\right)]$/ in an Arrhenius plot with an activation energy of $E_{a}$ = 8 kJ/mol. The influence of relative humidity on $J_{\mathrm{PL}}^{dev}$ seems marginal. One may associate the Scott Blair element with the load-induced quasi-instantaneous re-arrangement of polymer network chains (characteristic time in the order of seconds).

**Figure 9:**
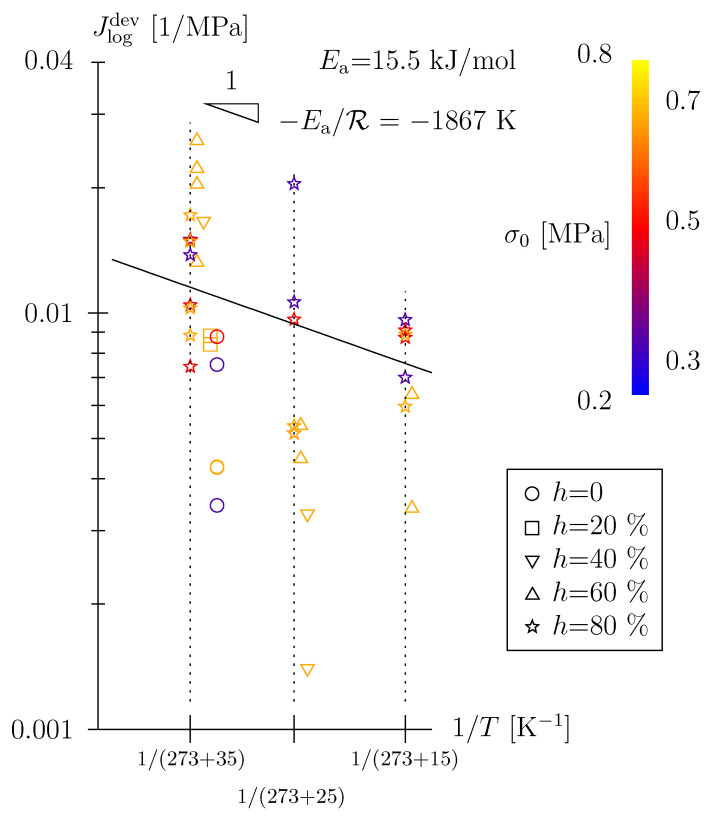
Arrhenius diagram of back-calculated Lomnitz creep compliance parameter $J_{log}^{dev}$; activation energy (trend line) was determined for *h* = 80% data only, $r^{2}$ = 0.20 (for better readability *h* < 80% data were slightly offset to the right).

For a certain threshold, i.e., with increasing temperature and/or increasing physisorbed water content (associated to *h*)—see Table 2—the Lomnitz element is activated. This may reflect:

- A load-induced breakage of carbamate H-bonds in the dry state (see Figure 1a, high temperature + low humidity) and a rearrangement of these H-bonds (no physisorbed water involved);
- A breakage of physisorbed water H-bonds (see Figure 1b–d, high humidity, all investigated temperatures), a “microdiffusion” to potential bonding sites and a re-establishment of urethane–water–urethane H-bonds. This breakage/microdiffusion/rearrangement process is characterized by $E_{a}$ = 15.5 kJ/mol (see Figure 9), which is significantly larger than $E_{a}$, characterizing a quasi-instantaneous, Scott Blair-type material response.

## 7. Summary and Concluding Remarks

The main findings of this paper can be summarized as follows: (i) The time-dependent behavior of TPUs under moderate loads may indeed be characterized in the realm of linear viscoelasticity. (ii) Hereby, a Scott Blair element is highly suitable to represent the short-term or quasi-instantaneous deformation. This power-law-type constitutive model with a low-value exponent (in the order of 0.06) may be better suited than the standard elastic model, the Hookean spring; the quasi instantaneous response is characterized by an Arrhenius type temperature dependency with $E_{a}$ = 8 kJ/mol (see Figure 8) and may be associated with the load-induced, quasi-instantaneous re-arrangement of polymer network chains. (iii) For higher temperatures and/or higher physisorbed water contents (equilibrium state associated with certain relative humidity), a secondary viscoelastic mechanism comes into play, well described by a Lomnitz element with a characteristic time of several minutes. Lomnitz-type behavior is characterized by a temperature dependency with $E_{a}$ = 15.5 kJ/mol and may be associated with the initiation of hydrogen-bond breakage and rearrangement (carbamate H-bonds and physisorbed water H-bonds).

With regard to the objectives stated in the Introduction, one may state that the latter have been addressed as follows: We assigned the probably most appropriate viscoelastic elements within the investigated range of experimental conditions (range for temperature and relative humidity (i.e., water content), level of loading) with an exceptionally sound representation of the experimental data (see Figure 6b and Figure 7). Furthermore, with a very low number of free, adjustable material parameters—two for the Scott Blair element or springpot, $J_{\mathrm{PL}}^{dev}$ and $n_{\mathrm{PL}}$, respectively, and four for the Scott Blair element connected in series with a Lomnitz element, $J_{\mathrm{PL}}^{dev}$, $n_{\mathrm{PL}}$, $J_{log}^{dev}$, and $\tau_{log}$, respectively—this opened the possibility to assign obvious microstructural mechanisms to the coherent constitutive elements (quasi-instantaneous response and secondary viscoelastic mechanism stated above). Along with the findings corresponding to the quantitative physisorption and vapor transport modeling presented in [4], the viscoelastic model proposed in this paper may advance the quantitative engineering modeling of the thermo-hygral-mechanical behavior of TPUs and TPU-based composite materials, allowing for the prediction of durability and long-term stability in industrial and structural applications.

## Figures and Tables

**Table 1:** Equilibrium physisorbed water content associated with sample enclosing humidity according to Figure 3 in [4].

Sample Enclosing Humidity *h* (%)	Water Content (–)
0	0
20	0.0025
40	0.0053
60	0.0091
80	0.0135

**Table 2:** Observed creep behavior dependent on environmental conditions. SB: Scott Blair element only. SB + L: Scott Blair element in series with Lomnitz element.

	*T* = 35 ${}^{\circ }$C	*T* = 25 ${}^{\circ }$C	*T* = 15 ${}^{\circ }$C
*h* = 0	SB + L	SB	SB
*h* = 20%	SB + L	no data	no data
*h* = 40%	SB + L	SB + L	SB
*h* = 60%	SB + L	SB + L	SB + L
*h* = 80%	SB + L	SB + L	SB + L

## Data Availability

Data will be made available upon request.

## Appendix A

### Appendix A.1. Laplace–Carson Transformation

The Laplace–Carson transformation of a function f(t) is given as

(A1) $$L\left[f\left(t\right)\right]=f^{★}\left(p\right)=p\int_{0}^{\infty }f\left(t\right)e^{-pt}dt.$$

The inverse Laplace–Carson transformation is defined in the complex plane as

(A2) $$L^{-1}\left[f^{★}\left(p\right)\right]=f\left(t\right)=\frac{1}{2i\pi }\int_{\Omega }\frac{f^{★}\left(p\right)}{p}e^{pt}dp,$$

where Ω is a parallel to the imaginary axis having all poles of $f^{★}\left(p\right)$ to the left.

### Appendix A.2. Mechano-Sorptive Coupling Effects Relevant in Creep Experiments

The Picket paradox, as summarized in [27], is stated as follows: “A previously dried concrete (sample #1) exhibits practically no creep. In the case of a concrete in which any exchange of moisture during loading is prevented (basic creep), the more evaporable water it contains, the more it creeps (sample #2). On the other hand, a concrete that dries during loading (drying creep) creeps even more (sample #3), although it changes gradually from the hydric state of (sample #2) to the hydric state of (sample #1).” Hence, with regard to creep deformation: (sample #3) > (sample #2) > (sample #1).

## Appendix B

See Table A1 and Table A2 for a summary of back-calculated material parameters.

**Table A1:** Environmental conditions, stress held constant during creep experiment, and back-calculated creep parameters.

*h* (%)	*T* (${}^{\circ }$C)	$\sigma_{0}$ (MPa)	$J_{\mathrm{PL}}^{\mathrm{dev}}$ (MPa${}^{-1}$)	$n_{\mathrm{PL}}$	$J_{\log}^{\mathrm{dev}}$ (MPa${}^{-1}$)	$\tau_{\log}$ (s)
0	15	0.3	0.1065	0.05550		
0	15	0.3	0.1030	0.05390		
0	15	0.3	0.1124	0.05624		
0	15	0.5	0.1498	0.06795		
0	15	0.5	0.1197	0.06134		
0	15	0.7	0.1281	0.06621		
0	15	0.7	0.1191	0.06315		
0	25	0.3	0.1095	0.04975		
0	25	0.3	0.1127	0.05599		
0	25	0.5	0.1126	0.05015		
0	25	0.5	0.1131	0.04860		
0	25	0.7	0.1199	0.05476		
0	25	0.7	0.1185	0.05707		
0	35	0.3	0.1235	0.04822	0.003452	44.0
0	35	0.3	0.1294	0.05455	0.007519	116.1
0	35	0.5	0.1552	0.05518	0.008770	188.9
0	35	0.5	0.1192	0.05921		
0	35	0.7	0.1614	0.06720	0.004256	380.8
0	35	0.7	0.1756	0.06980	0.004270	302.4
20	35	0.7	0.1526	0.06933	0.008408	570.1
20	35	0.7	0.1420	0.07189	0.008813	398.2
40	15	0.7	0.1113	0.06461		
40	15	0.7	0.1045	0.05176		
40	25	0.7	0.1184	0.05773	0.003291	86.7
40	25	0.7	0.1227	0.05657	0.001401	68.7
40	35	0.7	0.1454	0.05723	0.016617	693.6
60	15	0.7	0.1322	0.06342	0.003394	645.0
60	15	0.7	0.1193	0.07409		
60	15	0.7	0.1525	0.06356	0.006377	142.2
60	25	0.7	0.1516	0.06955	0.004467	82.2
60	25	0.7	0.1276	0.05978	0.005368	213.1
60	35	0.7	0.1787	0.07364	0.022216	565.7
60	35	0.7	0.1738	0.07191	0.025955	516.9
60	35	0.7	0.1491	0.07481	0.013237	399.8
60	35	0.7	0.1664	0.07116	0.020380	629.0

**Table A2:** Environmental conditions, stress held constant during creep experiment, and back-calculated creep parameters (cont’d).

*h* (%)	*T* (${}^{\circ }$C)	$\sigma_{0}$ (MPa)	$J_{\mathrm{PL}}^{\mathrm{dev}}$ (MPa${}^{-1}$)	$n_{\mathrm{PL}}$	$J_{\log}^{\mathrm{dev}}$ (MPa${}^{-1}$)	$\tau_{\log}$ (s)
80	15	0.3	0.1242	0.05172	0.009627	119.2
80	15	0.3	0.1228	0.04807	0.007002	169.3
80	15	0.5	0.1150	0.04642	0.009094	438.3
80	15	0.5	0.1138	0.04872	0.008729	571.5
80	15	0.7	0.1204	0.05780	0.005961	319.9
80	15	0.7	0.1189	0.05426	0.008882	460.2
80	25	0.3	0.1501	0.05526	0.020429	147.9
80	25	0.3	0.1328	0.05084	0.010625	105.8
80	25	0.5	0.1348	0.04695	0.005140	123.8
80	25	0.5	0.1291	0.05033	0.009656	156.1
80	25	0.7	0.1360	0.05217	0.005354	82.1
80	25	0.7	0.1391	0.05197	0.005134	152.6
80	35	0.3	0.1382	0.04498	0.013790	240.3
80	35	0.3	0.1364	0.04833	0.015013	250.5
80	35	0.5	0.1393	0.04506	0.010454	481.9
80	35	0.5	0.1473	0.05515	0.014963	246.0
80	35	0.5	0.1425	0.05226	0.007438	128.9
80	35	0.7	0.1423	0.05055	0.010274	392.2
80	35	0.7	0.1538	0.05272	0.008839	291.3
80	35	0.7	0.1594	0.06704	0.017200	544.9
80	35	0.7	0.1552	0.07142	0.014856	403.7
